# The orphan adapter protein SLY1 as a novel anti-apoptotic protein required for thymocyte development

**DOI:** 10.1186/1471-2172-10-38

**Published:** 2009-07-15

**Authors:** Bernhard Reis, Klaus Pfeffer, Sandra Beer-Hammer

**Affiliations:** 1 Institute of Medical Microbiology and Hospital Hygiene, Heinrich-Heine-University Duesseldorf, Universitaetsstrasse 1, D-40225 Duesseldorf, Germany; 2 Institute of Experimental and Clinical Pharmacology and Toxicology, Eberhard-Karls-University Tuebingen, Wilhelmstrasse 56, D-72074 Tuebingen, Germany

## Abstract

**Background:**

SH3 containing Lymphocyte Protein (SLY1) is a putative adapter protein exclusively expressed in lymphocytes which is involved in antigen receptor induced activation. We previously have generated SLY1^Δ/Δ ^mice harbouring a partial deletion in the N-terminal region of SLY1 which revealed profound immunological defects in T and B cell functions.

**Results:**

In this study, T cell development in SLY1^-/- ^and SLY1^Δ/Δ ^mice was analysed *ex vivo *and upon cultivation with the bone marrow stromal cell line OP9. SLY1-deficient thymocytes were compromised in inducing nutrient receptor expression and ribosomal protein S6 phosphorylation, indicating a defect in mTOR complex activation. Furthermore, SLY1 was identified as a novel anti-apoptotic protein required for developmental progression of T cell precursors to the CD4^+^CD8^+ ^double-positive stage by protecting from premature programmed cell death initiation in developing CD4^-^CD8^- ^double-negative thymocytes. In addition, SLY1 phosphorylation was differentially regulated upon Notch ligand-mediated stimulation and expression of the preTCR.

**Conclusion:**

Thus, our results suggest a non-redundant role for SLY1 in integrating signals from both receptors in early T cell progenitors in the thymus.

## Background

T cell development mainly takes place in the thymus and is characterized by defined developmental stages ultimately leading to the generation of mature αβ and γδ T cells. The earliest T cell progenitors entering the thymus via the bloodstream lack CD4 and CD8 expression and are therefore called double-negative (DN). DN thymocytes can be further subdivided into four successive developmental stages according to the expression of CD25 (IL-2 receptor α chain) and CD44 (Pgp-1) [1,2]. The most immature progenitors (DN1) are assigned to the CD44^+^CD25^- ^subset. Upregulation of CD25 marks progression to the DN2 stage (CD44^+^CD25^+^). Irreversible commitment to the T cell lineage is not established until the DN3 stage, which can be identified as the CD44^-^CD25^+ ^DN subset. In this cell population, rearrangement of the TCRβ- or TCRγδ genes is accomplished, leading to either development into the αβ- or the γδ lineage. For αβ T cells, a successfully generated TCRβ chain leads to formation of the preTCR via pairing with the preTα chain and CD3 signalling complexes. Thymocytes failing to generate a preTCR undergo programmed cell death [3]. Thymocytes otherwise destined to induce programmed cell death are rescued from apoptosis upon preTCR generation. This event is termed β-selection, leading to extensive cellular expansion and cessation of further TCRβ locus recombination (allelic exclusion). Cells which have passed this developmental checkpoint downregulate CD25 to become DN4 cells and then proceed to the numerically dominant CD4and CD8 double-positive (DP) stage. Lymphocytes evading cell death are frequently the origin of malignant transformations [4,5]. Although of high clinical relevance, the regulation of pro- and anti-apoptotic processes at this specific stage of development is still incompletely understood.

In the past years, it has been established that for commitment to the T cell lineage Notch signalling is essential [6-9]. In combination with the preTCR, Notch induces proliferation and differentiation to DP thymocytes [10,11]. More recently, Notch ligand engagement was additionally shown to be crucial for controlling progenitor cell metabolism by activating Phosphatidyl-Inositol-Kinase-1 (PDK-1), Akt and the mammalian Target of Rapamycin (mTOR) complex [12,13]. Notch- and preTCR-dependent upregulation of mTOR induces expression of the nutrient receptors CD71 (transferrin receptor) and CD98 (amino acid transporter). This is, together with the PDK-1- and mTOR-dependent activation of AGC-serine kinases like S6 Kinase 1 and Ribosomal S6 Kinase, a key event for cell mass increase and induction of a proliferation-competent status, eventually leading to six to ten consecutive rounds of cell division after passage of the β-selection checkpoint.

SH3 Lymphocyte Protein (SLY1) is a putative adapter protein containing a SH3- and SAM-domain. It constitutes the first described member of a family of highly homologous proteins conserved in mammals whose molecular function is still elusive. The protein SLY1 consists of 380 amino acids and is exclusively expressed in lymphocytes [14]. It has been originally found in an adhesion assay screen using a T cell lymphoma cDNA library. SLY1 has been independently identified by another group during a screen for new serine kinase substrates [15]. There it was shown that SLY1 is specifically phosphorylated upon T cell receptor (TCR)-triggering or stimulation with phorbol esters at Serin27. This phosphorylation could be prevented by Protein Kinase C (PKC)- or PI3Kinase-inhibitors, however, the directly phosphorylating kinase has not been identified to date. Further hints for a possible involvement of SLY1 in the TCR-transduction pathway were delivered by the immune-compromised phenotype of mice expressing a truncated SLY1 protein. This truncation resulted in aberrant subcellular localisation of the remaining protein SLY1Δ which is lacking part of the bipartite nuclar localisation sequence and the known phosphorylation site [16]. Furthermore, it was shown that SLY1 plays a substantial role in the development and activation of immune cells, although the underlying mechanism of this phenotype could not be clarified [16,17].

Two additional highly homologous proteins have been described: SLY2, also termed SAMSN1/NASH1/HACS1, is more broadly expressed in haematopoietic tissues, endothelial cells and myeloid leukemias and myelomas [18,19]. SLY2 has been implicated in inducing a plasma-cell-like phenotype when overexpressed in B cells and has been linked to the inhibitory receptor PirB [20]. The *SLy2 *locus lies in a region which is frequently disrupted in translocation events leading to haematopoietic malignancies [18]. Most recent data suggest the involvement of SLY2 as tumour suppressor in human lung cancer [21]. SLY3 (SASH1) is expressed in almost all tissues and has been repeatedly described as a tumour suppressor based on its frequent downregulation in breast cancer and colon carcinoma [22,23].

In this study, mice harbouring a complete deletion of SLY1 protein were generated and thymocyte development was subsequently analysed. Thereby, an important role for the orphan adapter protein SLY1 at the thymocyte DN to DP progression was identified, leading to a reduction in thymic cellularity by about 50%. SLY1-deficient thymocytes were compromised in inducing nutrient receptor expression and ribosomal protein S6 phosphorylation, indicating a defect in mTOR complex activation and suggesting a role for SLY1 in integrating signals derived from the preTCR and from the Notch receptor. This defect resulted in abnormally increased deletion of precursor cells by induction of programmed cell death, yielding a strong reduction in DP thymocyte numbers. Interestingly, SLY1Δ mice expressing a protein harbouring a deletion of 81 amino acids in the N-terminal region of SLY1 exhibited an identical phenotype. Thus, a non redundant role for this region comprising the phosphorylation site and part of the nuclear localisation sequence (NLS) could be assigned.

## Results

**Figure 1:**
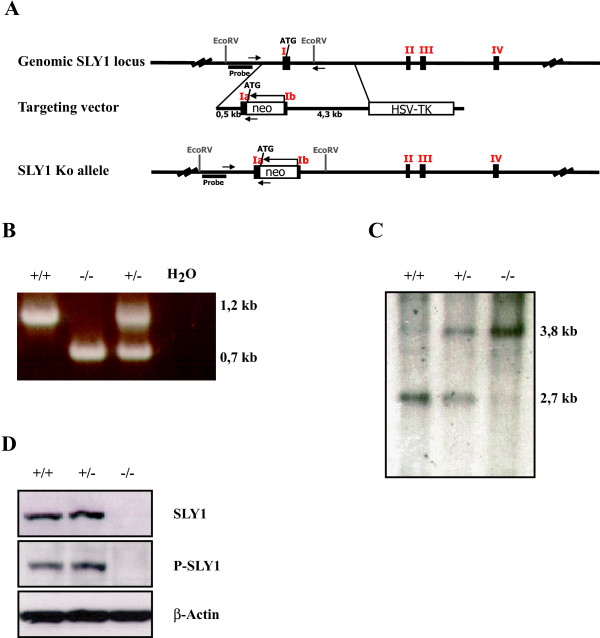
**Generation of SLY1 ko mice**. **A**. Scheme of the *sly1 *genomic locus, targeting vector and inserted *neomycin *cassette after homologous recombination. The *neomycin *cassette is inserted in inverted orientation directly after the starting ATG. **B**. Screening PCR with genomic tail DNA and primers as described in material and methods. **C**. Southern blot after digestion of genomic tail DNA with EcoRV and detection with ^32^P-labeled probe depicted in A. **D**. Western Blot assessing the successful inactivation of the *sly1 *gene using two different SLY1-specific antibodies and β-actin as loading control.

To elucidate the role of SLY1, mice expressing a truncated SLY1 protein (SLY1Δ) have been generated previously [16]. SLY1^Δ/Δ ^mice display a severe defect in lymphocyte numbers as determined by measuring cellularity of lymphoid organs and lymphocyte activation in a mixed lymphocyte reaction. As the expression of a truncated protein can display unpredictable results, the phenotype of SLY1^Δ/Δ ^mice may actually not reflect the precise molecular role of SLY1 in lymphocyte development and activation. Therefore, SLY1^-/-^-mice were generated by injection of E14 embryonic stem cells carrying a targeted homologous inactivation of SLY1. Figure 1A shows the arrangement of the first exons of the genomic *SLy1 *locus and of the targeting vector. After homologous recombination of the target vector with the *SLy1 *locus, a *neomycin *cassette is integrated in reverse orientation in exon 1. Arrows indicate the position of screening oligonucleotides which lead to a 1.2 kb versus 0.7 kb PCR product for the wildtype (wt) and knockout (ko) allele, respectively (Figure 1B). Germ line transmission of the ko allele was confirmed by Southern blotting (Figure 1C). Complete inactivation of the *SLy1 *gene was verified by Western blot (Figure 1D) using two different antibodies recognizing different epitopes of SLY1 protein.

### Reduced cellularity of lymphoid organs

**Figure 2:**
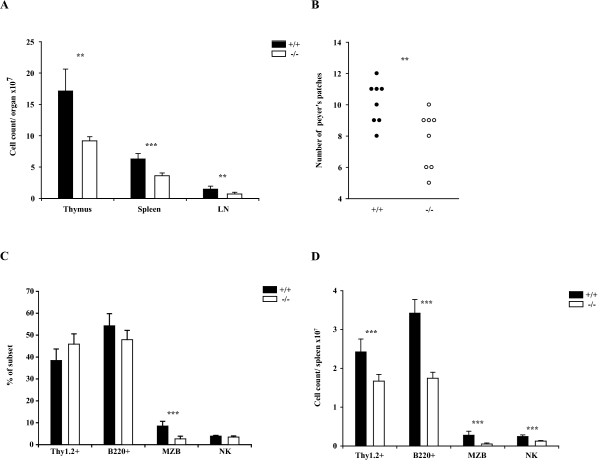
**Reduced cellularity of lymphoid organs in SLY1^-/-^-mice**. **A**. Mean cell count of thymus, spleen and pooled axial and inguinal lymph nodes was determined (n = 4). **B**. Reduced number of visible Peyer's patches in SLY1^-/-^-mice. **C**. FACS analysis of splenic lymphocyte subpopulations. T cells were determined as Thy1.2^+^, B cells as B220^+^, marginal zone B cells as CD21^high^CD23^- ^of B220^+^, and NK cells as CD3^-^CD56^+ ^(n = 9). **D**. Absolute cell counts/spleen (n = 9). **p ≤ 0.01; *** p ≤ 0.001.

SLY1^-/- ^mice were born at normal Mendelian ratios, appeared healthy and were fertile. SLY1^Δ/Δ ^mice have been reported to display diminished lymphoid organ cellularity [16]. To this regard, the impact of a complete SLY1-deficiency was assessed. Analysis of lymphoid organ cell count revealed a significant reduction of lymphoid organ cell numbers of SLY1^-/- ^mice compared to SLY1^+/+ ^littermates (Figure 2A). Cell numbers of thymi were reduced by 46%, splenocyte cellularity by 42% and lymph node cellularity by 51%. The mean number of macroscopically visible Peyer's patches also was clearly reduced to 7.8 +1.8 compared to 10.1 +1.4 in wt littermates (Figure 2B). Nevertheless, major SLY1^-/- ^splenic lymphocyte subpopulation ratios as determined by FACS were grossly normal except a reduction in percentages of marginal zone B cells (Figure 2C). Overall, all SLY1^-/- ^lymphocyte subpopulations were decreased in cell number due to the smaller organ size (Figure 2D), even though major B and T cell subpopulation ratios were normal. In summary, these data are strikingly reminiscent of the previously described phenotype of SLY1^Δ/Δ ^mice, which also displayed a reduction in cellularity of lymphoid organs, namely spleen, thymus, lymph nodes and Peyer's patches [16].

### Delayed T cell development

**Figure 3:**
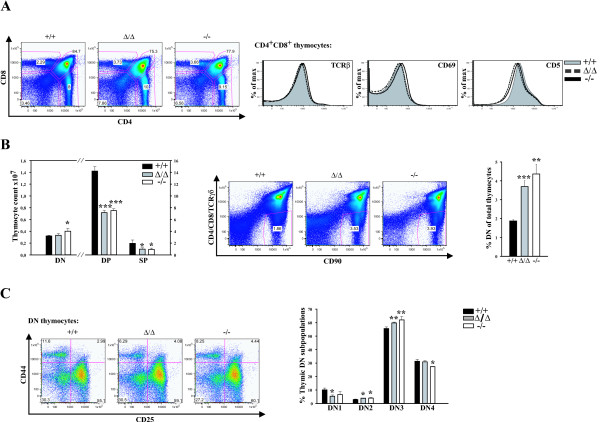
**Block in thymocyte CD4^-^CD8^- ^(DN) to CD4^+^CD8^+ ^(DP) differentiation**. **A**. Total thymocyte CD4- and CD8-expression was determined by FACS analysis (left panel). Histograms (right panel): Surface expression of TCRβ, CD69 and CD5 on gated CD4^+^CD8^+ ^thymocytes. **B**. Cell count of DN, DP or CD4- and CD8-single positive (SP) thymocytes (left panel). Gating (middle panel) and percentage of DN thymocytes relative to total thymocytes (right panel). **C**. DN thymocytes were gated as shown in B and a subset analysis according to expression of CD44 and CD25 was performed. DN1 = CD44^+^CD25^-^; DN2 = CD44^+^CD25^+^; DN3 = CD44^-^CD25^+^; DN4 = CD44^-^CD25^-^. Dot plots show representative data of three independent experiments (n = 3). *p ≤ 0.05; **p ≤ 0.001; *** p ≤ 0.0005.

As both partial and complete deletion of SLY1 protein had an obviously deleterious effect on thymocyte cell number leading to a reduction of about 50% of wt levels, SLY1-dependent thymocyte development was examined more closely. SLY1 is expressed in all major thymocyte subsets, as determined by RT-PCR (see Additional file 1). To gain a better understanding of the impact of the N-terminal truncation in the SLY1 protein versus complete deletion, SLY1^Δ/Δ ^thymocytes were included in the following experiments. CD4- and CD8-staining of total thymocytes revealed no apparent differences in percentages of DP and single-positive (SP) cells in the thymus (Figure 3A, left panel). Comparable expression of TCRβ, CD69 and CD5 on DP thymocytes indicated normal TCR signalling and positive selection events in SLY1-targeted thymocytes (Figure 3A, right panel). In line with the reduced thymocyte cellularity, SLY1^-/- ^and SLY1^Δ/Δ ^mice had markedly reduced total numbers of CD4^+ ^and CD8^+ ^DP and SP thymocytes, only a minor decrease in DN thymocyte counts was observed (Figure 3B, left panel). These results suggested a partially delayed development of DN to DP thymocytes in the absence of a functional SLY1 protein. In accordance with this hypothesis, a doubling in the percentage of DN cells in SLY1^-/- ^and SLY1^Δ/Δ ^thymi was observed (Figure 3B middle and right panel). This prompted us to further analyse the DN subset frequencies based on the expression of CD25 and CD44 (Figure 3C). We observed a slight decrease in DN1, a slight increase in DN2 and DN3 ratios in SLY1^-/- ^and SLY1^Δ/Δ ^thymi, and a slight decrease in DN4. Therefore, SLY1 appears to be required for developmental progression in all four thymic DN subsets and the block in development could not be assigned to a specific DN stage, providing a first hint that SLY1 expression is especially critical in thymic DN progenitor cells.

### Defect in proliferation and differentiation *in vitro*

**Figure 4:**
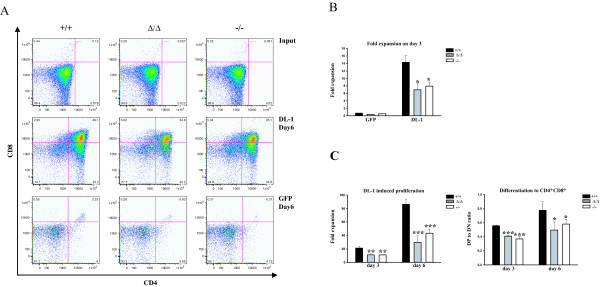
**SLY1-targeted DN thymocytes exhibit impaired proliferation and differentiation when cultured on OP9 DL-1 cells**. **A**. CD4 and CD8 FACS profiles of sorted DN thymocytes before cultivation (input, upper row), and after cultivation for six days on OP9 DL-1 (middle row) or on OP9 GFP control cells (lower row). **B**. DL-1-dependent expansion of DN thymocytes after three days culture. **C**. Fold expansion on day three and day six (left panel). Differentiation rate (right panel) corresponds to the percentage of DP cells divided by the percentage of remaining DN cells. The expansion rate was determined by counting triplicates and comparing to initial seeding numbers on day 0. Representative data of at least three independently performed experiments is shown. *p ≤ 0.05; **p ≤ 0.005; *** p ≤ 0.001.

The partial block in developmental progression of DN thymocytes lacking functional SLY1 protein indicated that proliferation of DN thymocytes in SLY1-defective mice might be impaired. To test this hypothesis, we analysed SLY1-dependent thymocyte proliferation and differentiation *in vitro*. To this end, the bone marrow stromal cell line OP9 was used allowing *in vitro *differentiation of DN to DP thymocytes induced by presentation of the Notch ligand Delta-like 1 (DL-1) on the surface of OP9 cells [6,24]. Congruent with published data, sorted DN thymocytes proliferated and upregulated CD4- and CD8-expression dependent on the presence of Notch ligand DL-1 after three and six days of cultivation on OP9 cells (Figure 4A, B and data not shown). As expected from previously observed *ex vivo *data, we found a markedly reduced expansion rate of sorted SLY1^-/- ^and SLY1^Δ/Δ ^DN thymocytes upon cultivation on OP9 cells compared to wt littermates (Figure 4B and 4C, left panel). Additionally, the differentiation of SLY1^-/- ^and SLY1^Δ/Δ ^DN thymocytes was diminished relative to littermate controls (Figure 4C, right panel). If sorted DN3 cells were used, differentiation was equally affected, also adding IL-7 to the medium gave similar results (see Additional file 2).

### Partially impaired activation of mTOR-complex in SLY1-defective DN thymocytes

**Figure 5:**
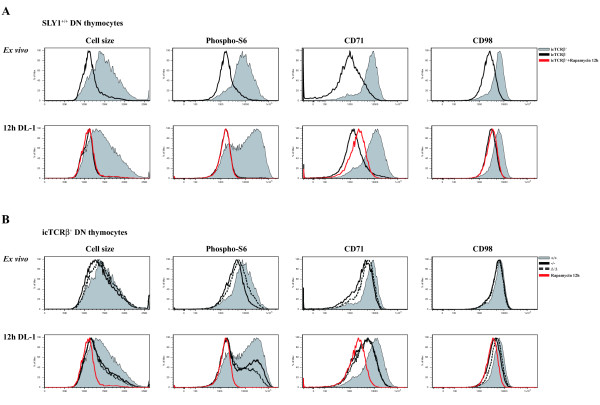
**mTOR-signalling is diminished in developing SLY1^-/- ^and SLY1^Δ/Δ ^DN thymocytes**. Cell size, activation of S6 kinase and expression levels of the nutrient receptors CD71 and CD98 were determined as surrogate markers for mTOR activity in thymocytes. Phosphorylation of ribosomal protein S6 was used as a marker of S6 Kinase activity [25]. **A**. SLY1^+/+ ^DN thymocytes were analysed *ex vivo *and after 12 h culture on OP9 DL-1 cells. Thymocytes were further subdivided by gating on preTCR-negative and -positive cells. **B**. Activation of mTOR in preTCR-expressing SLY1^+/+^, SLY1^-/- ^and SLY1^Δ/Δ ^thymocytes was analysed *ex vivo *and after OP9 DL-1 culture *in vitro *for 12 hours. 20 nM Rapamycin was added as a control during the cultivation period to inhibit mTOR activation. Histograms show representative data of four independent experiments (n = 4).

One of the cellular outcomes of DN thymocytes successfully passing the β-selection check point is the upregulation of metabolic activity. This is a prerequisite to cope with the increasing energy demands at this developmental stage needed to allow for the upcoming extensive proliferation and differentiation of DN to DP thymocytes. Of particular importance in this context is the activation of the Rapamycin-sensitive mTOR-complex eventually resulting in an increase in cell mass and subsequent induction of cell division. A first hint for a blunted mTOR-dependent activation in SLY1^-/- ^and SLY1^Δ/Δ ^thymocytes came from the observation of a reduced frequency of thymocytes with increased cell size *ex vivo *and after OP9 DL-1 culture (see Additional file 3 and Figure 5B left panel)[25]. Several key targets of mTOR in activated DN thymocytes have been identified so far, their activation or induced expression being strictly dependent on the combined engagement of a successfully rearranged preTCR and Notch receptor [11-13]. Well reported events at this developmental stage are the activation of S6 Kinase and upregulation of the nutrient receptors CD71 and CD98, all of which have been shown to be Rapamycin-sensitive [13]. First, mTOR activation in SLY1^+/+ ^thymocyte T cell precursor populations *ex vivo *and after cultivation for 12 h on OP9 DL-1 cells was analysed. This was accomplished by determination of cell size, phosphorylation of ribosomal protein S6, CD71 and CD98 expression as surrogate markers for mTOR activity (Figure 5A). In line with published data, activation of mTOR in DN thymocytes is absolutely dependent on preTCR-expression *ex vivo *and *in vitro*. Furthermore, mTOR activation strictly requires Notch signalling (see Additional file 3) and can be inhibited by addition of Rapamycin *in vitro*. Next, this single-cell based analysis was extended to SLY1^-/- ^and SLY1^Δ/Δ ^thymocytes *ex vivo *and after 12 h culture on OP9 DL-1 cells (Figure 5B). In summary, the activation status of mTOR in preTCR-positive DN thymocytes of SLY1^-/- ^and SLY1^Δ/Δ ^mice was clearly reduced compared to wt littermates under both conditions tested. The activation of mTOR in the absence of Notch signalling or in preTCR-negative thymocytes was equally low (see Additional file 3 and data not shown). These results suggest a defect in inducing mTOR-mediated activation signals in a subset of T cell precursor cells in SLY1^-/- ^and SLY1^Δ/Δ ^mice in response to developmental needs. Moreover, we propose a defect in transmitting signals from either the preTCR or the Notch receptor to the mTOR protein complex.

### Increased apoptosis induction in SLY1-defective DN3 thymocytes

**Figure 6:**
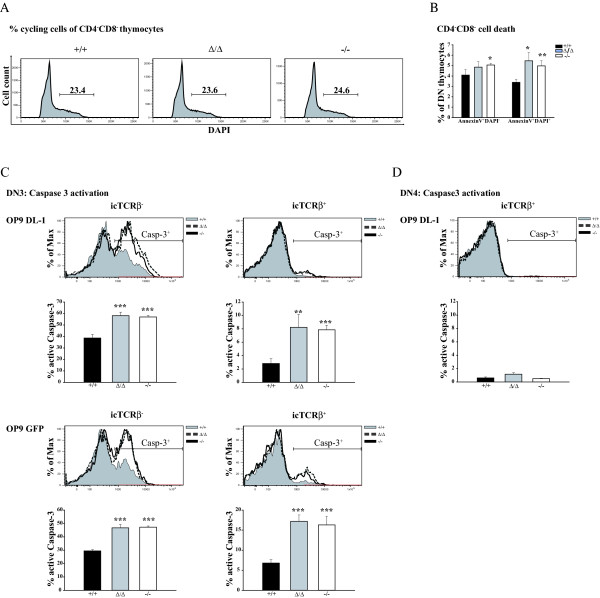
**Increased apoptosis of OP9 cultured DN thymocytes in the absence of functional SLY1 protein**. **A**. Cell cycle analysis of permeabilized DN cells after 2 days of OP9 DL-1 culture using DAPI. **B**. AnnexinV and DAPI staining of DN thymocytes cultivated for 4 days on OP9 DL-1 cells. **C**. SLY1-targeting results in increased Caspase-3 activation independent of TCRβ-expression and Notch signalling. Sorted DN3 thymocytes were cultured on OP9 cells for 2 days and then Caspase-3 active fragment in DN cells was analysed dependent on TCRβ-expression. **D**. Intracellular Caspase-3 activation in DN4 cells is negligible. *p ≤ 0.05; **p ≤ 0.01; *** p ≤ 0.001.

A decrease in the expansion rate of proliferating cells can be either mediated by alterations in cell cycle progression or elevated apoptosis induction, or even both. To identify the underlying mechanism for the observed proliferation defect, sorted DN thymocytes were plated on OP9 DL-1 cells and cell cycle distribution as well as apoptosis induction was assessed. We found an equal cell cycle distribution based on DNA-staining of permeabilized cells with DAPI (Figure 6A). Yet an increase in early (AnnexinV^+^DAPI^-^) and late (AnnexinV^+^DAPI^+^) apoptosis induction in SLY1-targeted thymocytes was detected (Figure 6B). Therefore we hypothesized that the impaired proliferation can be attributed to an increased apoptosis rate in the absence of functional SLY1 protein. PreTCR-expression and Notch signalling have both been reported to play pivotal roles in protecting DN thymocytes from apoptosis at the β-selection checkpoint [10,12,26]. Interestingly, SLY1 protein has been reported to be specifically phosphorylated after TCR crosslinking in peripheral T cells [15]. It was hence reasonable to speculate a role for SLY1 downstream of the preTCR or Notch in this context. To dissect the pathways SLY1 is involved in, we cultivated sorted DN3 cells on OP9 DL-1 and OP9 control cells to discriminate the role of Notch signalling, and used Caspase-3 active fragment as an intracellular marker for apoptosis induction (Figure 6C). Intracellular staining for TCRβ-expression served to identify cells which had successfully rearranged their TCR-β-chain and which should therefore receive preTCR-derived proliferative and survival signals. Intracellular TCRβ-expression was similar between wt cells and SLY1-targeted cells (data not shown). As anticipated, Caspase-3 activation was lowest in wt preTCR-positive DN3 thymocytes cultivated on OP9 DL-1 cells, thus receiving a preTCR-derived and a Notch-derived pro-survival signal, accounting for approximately 2% of Caspase-3 active fragment positive thymocytes (Figure 6C, histogram overlay and diagram upper right panel). In this setting, which physiologically corresponds to thymocytes successfully passing β-selection, apoptosis induction was increased 4-fold in SLY1^-/- ^and SLY1^Δ/Δ ^DN3 thymocytes compared to wt littermate controls. In contrast, thymocytes failing to rearrange the TCRβ-chain stay TCRβ-negative and hence are increasingly prone to programmed cell death initiation. This was indeed the case: apoptosis induction in TCRβ-negative wt DN3 thymocytes was 20 times higher than in their TCRβ-positive counterparts (compare wt apoptosis upper left with upper right panel). Again, the percentage of Caspase-3 positive progenitor cells was markedly increased (by 20%) in SLY1^-/- ^or SLY^Δ/Δ ^cultures. Interestingly, SLY1^-/- ^and SLY^Δ/Δ ^thymocytes also displayed increased susceptibility towards programmed cell death initiation in the absence of Notch signalling (lower part of Figure 6C). Yet wt TCRβ-negative DN3 thymocytes cultivated on OP9 control cells were highly susceptible to apoptosis, accounting for around 30% Caspase-3-positive cells. Though the rate of apoptotic SLY1^-/- ^and SLY1^Δ/Δ ^DN3 thymocytes was elevated roughly by 15% relative to wt controls (left part, lower panel). We also observed a more than twofold increase in Caspase-3 activation in the preTCR-positive population of DN3 thymocytes when plated on OP9 control cells in the absence of SLY1. Thus, an overall increase in programmed cell death was observed in both SLY1^-/- ^and SLY1^Δ/Δ ^DN3 thymocytes. In general, TCRβ-expression was the major pro-survival determinant, Notch signals even were detrimental if thymocytes failed to successfully rearrange their TCRβ-genes (compare left part upper and lower panel). Nevertheless, Caspase-3 activation was elevated in SLY1^-/- ^and SLY1^Δ/Δ ^DN3 thymocytes independent of the availability of signals from the preTCR or the Notch receptor or even both. In summary, these results suggest a general anti-apoptotic role of SLY1 in DN3 thymocytes. Remarkably, programmed cell death in DN4 cells was equally undetectable independent of SLY1-expression (Figure 6E). This data supports a specific role for SLY1 in protecting DN3 thymocytes during the β-selection checkpoint. To further elucidate a possible role for SLY1 in amplifying Notch-derived signals, a titration of γ-secretase-inhibitor (GSI) which is reported to inhibit specifically Notch receptor activation on sorted DN3 SLY^+/+^, SLY1^-/- ^and SLY1^Δ/Δ ^thymocytes was performed (see Additional file 4). After six days of cultivation on OP9 cells, differentiation and proliferation was assessed. Appearance of DP cells gradually decreased with increasing GSI concentrations likewise did the expansion rate. At 1 μM GSI, inhibition was almost complete, as assessed by comparing the proliferation and differentiation rate with the OP9 GFP cultivated controls. SLY1^Δ/Δ ^and SLY1^-/- ^thymocytes were found to be more sensitive than SLY1^+/+ ^thymocytes to intermediate concentrations of GSI, supporting a function for SLY1 in signal propagation from the Notch receptor. However, this increased sensitivity was only very weakly pronounced and could also be attributed to a defect in integration of preTCR and Notch signals, as both signals are required for proliferation.

### Regulation of phosphorylation of SLY1 in thymocytes

**Figure 7:**
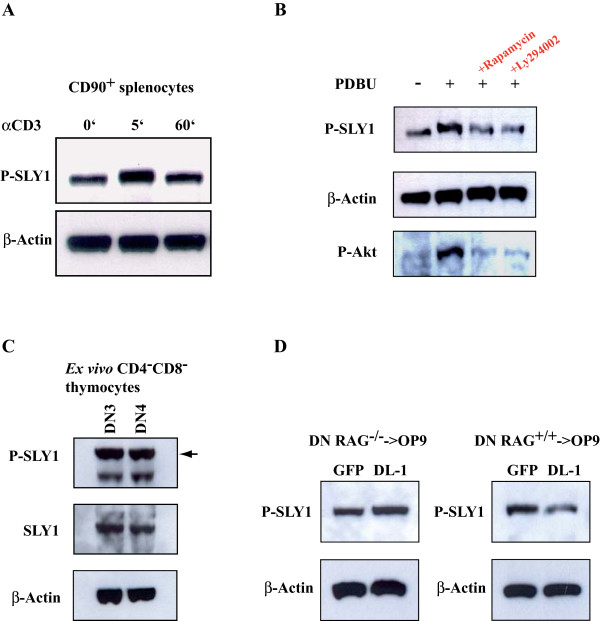
**Regulation of SLY1 phosphorylation in thymocytes**. **A**. Phosphorylation of SLY1 at serine27 in peripheral splenocytes. CD90^+ ^MACS-isolated T cells were stimulated with αCD3 (10 μg/ml) for the indicated time and analysed by Western blotting. **B**. Thymocytes were left unstimulated or incubated for 20' with 20 ng/ml PDBU. Where indicated, cells had been pretreated with Rapamycin (20 nM) or Ly294002 (10 μM) for 1 hour. **C**. DN3 and DN4 thymocytes were single-cell-sorted according to the expression of CD90, CD25 and CD44, lysed and analysed by Western blotting. **D**. RAG1^-/- ^or RAG1^+/+ ^DN3 thymocytes were plated over night on OP9 cells. DN thymocytes were then sorted by single-cell flow cytometry, lysed and analysed by Western blotting.

It has been reported that SLY1 is phosphorylated at serine27 specifically upon TCR-stimulation in human peripheral T-lymphocytes and not upon chemokine- or cytokine-driven stimulation [15]. This data implicated a function of SLY1 downstream of the TCR. In murine splenocytes, TCR-stimulation-induced phosphorylation can also be observed, albeit there is considerable basal phosphorylation in contrast to the situation in human lymphocytes (Figure 7A). Likewise, SLY1 phosphorylation in murine thymocytes is sensitive to inhibition by the PI3K inhibitor Ly294002 (Figure 7B). We also observed inhibition of SLY1-phosphorylation by treatment with Rapamycin, which has not been previously reported in peripheral lymphocytes and might reflect a differential regulation in thymocytes (Figure 7B). Interestingly, we detected a high degree of basal phosphorylation of SLY1 in early developing DN3 and DN4 thymocytes isolated *ex vivo*, suggesting a requirement of the phosphorylated variant of SLY1 in T cell development (Figure 7C). In DN3 thymocytes, the ratio of preTCR-expressing thymocytes is low and also gated preTCR-negative DN thymocytes exhibited increased apoptosis induction (Figure 6C, left panel), arguing against a role downstream of the preTCR. Therefore, we reasoned that SLY1 might be independently phosphorylated of preTCR-mediated signalling in early thymocytes. To test this hypothesis, RAG1^-/- ^DN thymocytes unable to rearrange the TCR-genes and therefore lacking preTCR-signalling were incubated over night on OP9 cells, sorted and then SLY1-phosphorylation was detected via Western blotting (Figure 7D, left panel). Indeed, we observed preTCR-independent phosphorylation of SLY1 in DN thymocytes as anticipated and in line with previous data. In addition, we detected an increase in SLY1 phosphorylation in RAG1^-/- ^thymocytes stimulated with Notch ligand. Unexpectedly, this Notch-induced increase in phosphorylation was only observed in preTCR-deficient thymocytes. In preTCR-sufficient RAG1^+/+ ^DN thymocytes, SLY1 phosphorylation was diminished upon Notch signalling (Figure 7D, right panel). This data suggests a role for SLY1 in integrating signals derived from the preTCR and from the Notch receptor leading to prevention of premature apoptosis initiation in developing thymocytes.

## Discussion

T cell development is a tightly regulated process involving numerous successive steps of differentiation. Of particular importance for the maturation of αβ T cells is the successful rearrangement of the VDJ genes coding for the TCRβ chain which then pairs with the preTα chain to initiate preTCR signalling [1,27-29]. In the present work, induction of programmed cell death in DN3 thymocytes could be precisely recapitulated as being principally dependent on intracellular preTCR expression, thereby supporting preTCR-signalling as the major decisive constraint in death versus survival decision. Furthermore, the orphan adapter protein SLY1 was identified as a novel anti-apoptotic protein required for developmental progression of T cell precursors to the DP stage. This anti-apoptotic function resulted in protection of thymocytes from premature programmed cell death initiation at the DN3 thymocyte stage. As a consequence, thymocyte DP number and therefore total thymus cellularity was severely reduced in SLY1-targeted mice.

Haematopoietic progenitors entering the thymus via the bloodstream depend on a sustained Notch receptor-ligand engagement to develop towards the T cell lineage [2,6,30,31]. The OP9 differentiation system can be used to recapitulate the signals required for haematopoietic precursor cells to undergo T cell development until the DP thymocyte stage *in vitro*. This differentiation system was used to further elucidate the underlying defect responsible for the reduced cellularity of SLY1^-/- ^and SLY1^Δ/Δ ^thymi. As anticipated from the *ex vivo *situation, a reduction in proliferation and differentiation upon OP9 culture relative to littermate controls was found. Thus, a migration defect of progenitor cells with a subsequent reduction in thymic repopulation capacity as the major cause for the resulting defect in thymic cellularity could be excluded. Furthermore, it could be clearly shown that the reduction in thymocyte proliferation is cell intrinsic and that the principal defect is not influenced by differences in thymic environment derived from stromal cells.

Yet Notch signalling is not only mandatory for commitment to the T cell lineage. Recently, it was also shown to be crucial for inducing a replication-competent status in thymocytes once preTCR signalling is initiated [10,12,13]. A prerequisite for cellular expansion is an increased metabolic activity including elevated expression of nutrient receptors and protein synthesis. The activity of mTOR has a key role in controlling this process. In peripheral T cells, growth factors have been reported to modulate Akt and mTOR activity and thereby to increase survival of T cells [32]. Growth factor withdrawal resulted in metabolic collapse and loss of nutrient receptor expression. In SLY1^-/- ^and SLY1^Δ/Δ ^thymocytes, induced expression of the nutrient receptors CD71 and CD98 as well as S6 Kinase activation were impaired relative to wt littermates. This diminished mTOR activity correlated with a reduced cell size increase. Therefore a defect in metabolic activity in SLY1-defective thymocytes can be inferred, leading to increased cell death rate and concomitant reduction of thymic cellularity.

Full mTOR activation in DN thymocytes is dependent on both Notch- and preTCR-derived signals [10,12,13]. There is limited insight into the signal integration process of both pathways in thymocytes. Current literature data suggests that signal integration occurs at the level of PI3Kinase activation. Notch signalling is thought to decrease expression of PTEN, the negative regulator of PI3Kinase activation [33]. preTCR-formation is thought to activate Akt, probably also via PI3Kinase activation [34]. It is not precisely clear how the presence of a preTCR without ligand engagement leads to the activation of Akt, and whether there are also more direct interactions of the Notch pathway with the PI3Kinase pathway which are not regulated by transcription. The present work may add insight into how the different signals are processed by connecting them with the orphan adapter protein SLY1. The reduced frequency of SLY1^-/- ^and SLY1^Δ/Δ ^progenitor cells with increased metabolic activity suggests a role for SLY1 in transmitting signals from either the preTCR or the Notch receptor to mTOR, or even for integrating signals from both receptors. As opposed to reports from peripheral T lymphocytes showing that SLY1 phosphorylation is strictly dependent on TCR-stimulation, we have shown in this study that SLY1-phosphorylation in thymocytes is independent of the preTCR. Interestingly, we have found that the phosphorylation of SLY1 at serine27 is differentially regulated upon Notch-stimulation dependent on the absence or presence of preTCR-signalling. This differing phosphorylation could be interpreted as a signal integration process of the two converging pathways downstream of the Notch receptor and downstream of the preTCR.

When cultured on OP9 DL-1 and OP9 GFP control cells, the anti-apoptotic function of SLY1 was independent of preTCR- and Notch-derived signals, indicating a general pro-survival role for SLY1 protein independent of both receptors. However, OP9 GFP cells have been reported to express mRNA for the Notch ligands *jagged-1 *and *jagged-2*, although in low amounts [6]. Therefore, it cannot be excluded that there might still be residual Notch-signals available to thymocytes cultivated on OP9 GFP control cells. In addition, OP9 cells secrete or present other unknown factors required by haematopoietic precursor cells. These residual signals induced by unknown factors could therefore transmit anti-apoptotic signals which are not properly transmitted in SLY1-defective thymocytes cultivated on OP9 GFP cells compared to the situation in SLY1^+/+ ^thymocytes. In addition, freshly isolated thymocytes have already been receiving Notch signals during their sojourn in the thymus *in vivo*, and therefore the increased apoptosis induction of SLY1-defective thymocytes on OP9 GFP cells compared to SLY1^+/+ ^thymocytes could already have been initiated in the thymus *in vivo *before isolation of the cells. Yet unexpected is the observation that Notch signalling can even drive thymocytes into apoptosis if they fail to generate a preTCR as observed upon OP9 culture of DN3 thymocytes in the present work. In contrast to this notice, Notch signalling in DN3 thymocytes is thought to play a general anti-apoptotic role by activating the Akt kinase pathway [12]. This study therefore adds additional complexity to the effector functions of Notch signalling in DN3 thymocytes, implying a pro-apoptotic outcome of Notch receptor activation in a certain cellular context.

As previously described, SLY1^Δ/Δ ^mice expressing a SLY1 protein harbouring a partial deletion in the N-terminal region show impaired immune responses [16,17]. The region missing in SLYΔ contains part of a bipartite nuclear localisation sequence and the known phosphorylation site closely associated to the potential localisation sequences. As a consequence, the subcellular localisation of SLY1Δ is shifted from a nucleocytoplasmic to a strictly cytoplasmic localisation [16]. The putative protein-protein interacting SH3- and SAM-domains remain intact in SLY1Δ, possibly still enabling its interference with other signalling proteins. However, a dominant negative effect of SLY1Δ can be excluded due to the comparable phenotype of SLY1^-/- ^mice. One conclusion that can be drawn from this data is that an essential function, at least for T cell development, of SLY1 is mediated by the amino acids at position 20–100. An apparent role for this deleted region could be either to regulate the correct subcellular localisation of the protein or to be directly required for interaction with SLY1 binding partners by providing a sequence specific recognition signal.

## Conclusion

In the present work, SLY1 is described as a novel anti-apoptotic protein required for thymocyte development by preventing DN thymocytes from premature initiation of programmed cell death. SLY1-negative thymocytes exhibited impaired activation of the mTOR complex, supporting an essential role of mTOR in thymocyte development. We therefore propose a role for SLY1 in signal integration of the Notch receptor and preTCR pathways culminating in the activation of the mTOR protein complex in developing thymocytes. As the *sly1 *gene is located on the X-chromosome in man, its function could be either negatively affected in some cases of x-linked immunodeficiency or activating mutations could contribute to lymphoma development. In the future, the identification of the interacting kinase and the precise molecular mechanism by which SLY1 contributes to mTOR activation and prevention of programmed cell death will be of great interest.

## Methods

### RNA isolation and RT-PCR

Total RNA from cells was isolated using Trizol Reagent (Invitrogen) according to the manufacture's instructions. First-strand cDNA synthesis was performed using 1 μg of total RNA with M-MLV reverse transcriptase and oligo dT primer (Invitrogen).

SLY1 transcripts were amplified with SLY1_exon6_forward: 5'-CCC GGA GGA TTC TGG GAA GA-3' and SLY1_exon8_reverse: 5'-GAA GTC AGT GTG GAC TCG GG-3'. The following primer sequences were used to amplify GAPDH: (forward) 5'-CAT GTA GGC CAT GAG GTC CAC CAC-3' and (reverse) 5'-TGA AGG TCG GTG TGA ACG GAT TTG GC-3'.

### Gene targeting and mice

Generation of SLY^Δ/Δ ^mice and genotyping has been described previously [16]. For generation of SLY^-/- ^mice, E14.1 embryonic stem (ES) cells from 129/Ola mice were grown in Dulbecco's modified Eagle's medium (Invitrogen, Carlsbad, CA, USA) supplemented with 2 mM glutamine (Seromed, Wien, Austria), leukemia inhibitory factor, 100 U/ml penicillin, 100 μg/ml streptomycin (Seromed), 50 μM 2-mercaptoethanol (Invitrogen) and 15% heat-inactivated fetal bovine serum (Pan Biotech, Aidenbach, Germany). Genomic fragments flanking the murine *sly1 *gene as well as *neomycin *and *thymidine kinase *were cloned into pBluescript (Agilent technologies, Santa Clara, CA, USA) and fully sequenced. The targeting vector was designed in such a way that the *neomycin *resistance cassette was inserted in reverse orientation in exon 1 of the *sly1 *gene 3' of the starting ATG without deleting any endogenous sequence. E14.1 ES cells were electroporated with the NotI-linearized targeting vector, and the transfected cells were subsequently subjected to G418 and ganciclovir selection. Clones carrying the correct homologous recombination were identified by Southern blot hybridization with the 5' flanking probe indicated in Figure 1 after digestion of ES cell DNA with EcoRV and BamHI. Single integration was verified by probing the Southern blot with the *neomycin *resistance cassette. Correctly targeted ES cell clones were injected into C57BL/6 blastocysts, which were transferred into pseudopregnant foster mice. Resulting chimeric mice were backcrossed to C57BL/6 mice, and germ line transmission of the targeted allele was again confirmed by Southern blot analysis. SLY1^-/- ^mice were backcrossed to the C57BL/6 background for six to ten generations. Wild-type littermates were used as controls. Genotyping of SLY1 knockout mice was performed by PCR with the following primers: 5'-TGA CGG CAG TAG GGA TGG TAG-3' (forward); 5'-CGC CTT CTT GAC GAG TTC TTC T-3' (neo reverse); 5'-AGT GGC CTG GGG GAG ATG T-3' (Wt reverse). SLY1^Δ/Δ ^mice were backcrossed for twelve generations.

Mice were kept according to national guidelines for animal care in an SPF animal facility. All animal work was performed according to the guidelines of the German national animal care regulations. RAG1^-/- ^mice were purchased from Jackson Laboratories.

### Flow cytometric analysis

Antibodies conjugated to fluorescein isothiocyanate, phycoerythrin, allophycocyanin, percp, phycoerythrin-cy7, allophycocyanin-cy7 and biotin were obtained from either Pharmingen (San Diego, CA, USA), Southern Biotech (Birmingham, Alabama, USA) or eBioscience (San Diego, CA, USA). Alexa-fluor488-conjugated anti-phospho-Akt and anti-phospho-S6 antibodies were obtained from Cell Signalling (Danvers, MA, USA). Cells were stained with saturating concentrations of antibody in accordance with the manufacturer's instructions. Data were acquired with either a FACSCalibur or FACSCanto (Becton Dickinson, Franklin Lakes, NJ, USA) using Cellquest or DIVA (Becton Dickinson) software and were analysed using Flowjo software (Treestar, San Carlos, CA, USA). Viable cells were gated according to their forward and sideward scatter profiles and DAPI (Invitrogen) exclusion. CD4 and CD8 DN Thy1.2^+ ^subsets were gated by lineage exclusion of CD4 and CD8 DP and SP cells and TCRγδ. DN subsets were further subdivided by CD25 and CD44 expression, defining CD25^-^CD44^+ ^as DN1, CD25^+^CD44^+ ^as DN2, CD25^+^CD44^- ^as DN3 and CD25^-^CD44^- ^as DN4, respectively. Cellular DNA content was measured by DAPI staining of methanol permeabilised cells. Phospho-S6 and phospho-Akt levels were assessed as described previously [11].

### OP9 cultures and assay

OP9 bone marrow stromal cells expressing Notch ligand Delta Like-1 (DL-1) and control OP9 cells [6] were a gift from Juan Carlos Zúñiga-Pflücker (Toronto, Canada). OP9 cells were maintained in α MEM (Invitrogen) supplemented with 50 μM 2-mercaptoethanol, 100 U/ml penicillin, 1 mg/ml streptomycin and 20% heat-inactivated FBS. Sorted DN thymocytes were co-cultured on OP9 DL-1 or OP9 control cells for the indicated time periods. DN thymocytes were obtained using an AutoMACS (Miltenyi Biotech, Bergisch-Gladbach, Germany) magnetic cell sorter by depleting CD4^+ ^and CD8^+ ^thymic subpopulations to greater than 97% purity. DN3 or DN4 cells were obtained by subsequently either positively sorting or depleting CD25-expressing DN3 cells, respectively. Alternatively, DN3 cells were obtained by sorting with a FACSAria single cell sorter (Becton Dickinson, Heidelberg, Germany) with greater than 98% purity. On the day of harvest thymocytes were filtered through 50 μm filters to remove OP9 cells before assessing developmental progression and proliferation of T lineage cells.

### Western blot analysis

Thymocytes or splenocytes were resuspended in lysis buffer containing 10 mMNaF, 1 mM sodium orthovanadate and protease inhibitor cocktail (Sigma) on ice for15 min. Lysates were separated by SDS-PAGE and transferred to nitrocellulose membranes (Perkin-Elmer, Rodgau-Jügesheim). After blocking with 5% milk in TBST, membranes were probed with primary antibodies and subsequently detected using horseradish peroxidase-linked goat anti-mouse or anti-rabbit IgG and visualized by the enhanced chemilumescent (ECL) detection system (GE Healthcare, Munich, Germany). Membranes were reprobed with anti-β-actin (Sigma, St. Louis, Missouri, USA). The anti-SLY1 antibody has been generated by Eurogentec (Seraing, Belgium) and has already been described [16]. Anti-phospho-SLY1 antibodies also have been generated by Eurogentec by immunizing rabbits with oligopeptide (H2N-LQR SSpS FKD FAK C-CONH2). Both antibodies have been tested extensively for specificity (data not shown).

### Statistical analyses

Statistical significance of differences between wildtype and SLY1-targeted mice were evaluated using the Students *t *test. *P *< 0.05 was considered significant. All results are presented as mean values ± SEM.

## Authors' contributions

BR has made substantial contributions to conception and design of the study, acquired, analysed, and interpreted the data, and drafted the manuscript. KP has given final approval of the version to be published. SB participated in the design and coordination of the study and helped to draft the manuscript. All authors read and approved the final manuscript.

## Supplementary Material

Additional file 1 **SLY1 is expressed in all major thymocyte subsets**. Semi-quantitative RT-PCR. Thymocytes were sorted according to their surface expression of CD4 and CD8. Subsequently, RNA was isolated and transcribed into cDNA. A serial 1:3 dilution of cDNA was used to amplify SLY1 or GAPDH transcripts with SLY1- or GAPDH-specific primers, respectively. Zipped folder containing EPS file. Click here for file

Additional file 2 **Proliferation and differentiation defect of sorted SLY1^-/- ^and SLY1^Δ/Δ ^DN3 thymocytes cannot be rescued by adding IL-7**. **A**. Fold expansion of sorted DN3 thymocytes after six days of culture on OP9 DL-1 cells. If indicated, 1 ng/ml mIL-7 was included in the cultures. **B**. Differentiation rate of sorted DN3 thymocytes after six days of culture on OP9 DL-1 cells without IL7. **C**. Differentiation rate of sorted DN3 thymocytes after six days of culture on OP9 DL-1 cells with 1 ng/ml murine IL7. Data are based on triplicate analyses. The differentiation rate corresponds to the percentage of DP cells divided by the percentage of remaining DN cells. The expansion rate was determined by comparing the obtained cell number on day six to initial seeding numbers on day 0. Representative data of two independently performed experiments is shown. Zipped folder containing EPS file. Click here for file

Additional file 3 **Notch- and preTCR-dependent activation of mTOR results in cell size increase and is diminished in sorted DN SLY1^-/- ^and SLY1^Δ/Δ ^thymocytes**. **A**. mTOR activation is strictly dependent on a combined preTCR- and Notch receptor-derived signal. Cell size of DN thymocytes was analysed after 12 h culture on OP9 GFP or OP DL-1 cells. Cells were gated on intracellular TCRβ-positive or -negative. **B**. Cell size overlay of DN SLY1^+/+^, SLY1^-/- ^and SLY1^Δ/Δ ^preTCR-positive thymocytes cultured on OP9 DL-1 cells. As a control, 20 nM Rapamycin was added during the cultivation period to inhibit mTOR activation. Zipped folder containing EPS file. Click here for file

Additional file 4 **γ-Secretase-inhibitor (GSI) titration of OP9-cultured DN3 cells**. Sorted SLY1^+/+^, SLY1^-/- ^and SLY1^Δ/Δ ^DN3 thymocytes were cultured on OP9 DL-1 or OP9 GFP cells for six days before analysis. Then, CD4- and CD8-expression was assessed by FACS (left panel). The expansion rate based on initial seeding numbers was determined (right panel). *p <0.01; **p <0.001; *** p <0.0001. Zipped folder containing EPS file. Click here for file
